# Ovarian carcinoid tumor with carcinoid heart syndrome: A case report and literature review

**DOI:** 10.1016/j.gore.2025.101997

**Published:** 2025-11-24

**Authors:** Minyoung Jang, Lakeisha Mulugeta-Gordon, Joseph Carver, Natalie Tupper, Julie Barbera, Lauren Schwartz, Nawar Latif

**Affiliations:** a Division of Gynecologic Oncology, Department of Obstetrics and Gynecology, University of Pennsylvania Perelman School of Medicine, 3400 Civic Center Boulevard, Philadelphia, PA 19104, USA; b Center for Cardio-Oncology, Abramson Cancer Center of the University of Pennsylvania, 3400 Civic Center Boulevard, Philadelphia, PA 19104, USA; c Department of Pathology and Laboratory Medicine, University of Pennsylvania Perelman School of Medicine, 3400 Spruce Street, Philadelphia, PA 19104, USA

**Keywords:** Ovarian carcinoid tumor, Carcinoid syndrome, Tricuspid regurgitation

## Abstract

**Background:**

Primary ovarian carcinoid tumors are rare neuroendocrine neoplasms comprising <0.1% of ovarian tumors and 1% of all carcinoid tumors. Up to 30% present with carcinoid syndrome, and approximately one-quarter of these develop carcinoid heart syndrome. Unlike gastrointestinal carcinoid tumors, ovarian lesions can cause carcinoid syndrome without hepatic metastases due to direct venous drainage into the systemic circulation.

**Case:**

A 46-year-old woman presented with abdominal discomfort, new onset hypertension, and lower extremity edema. Imaging revealed a 16.8 cm right adnexal mass with mixed solid and cystic components. Echocardiography demonstrated torrential tricuspid regurgitation related to thickened, immobile leaflets, consistent with carcinoid heart syndrome. Serum serotonin and chromogranin A were markedly elevated. She underwent exploratory laparotomy, total abdominal hysterectomy, bilateral salpingo-oophorectomy, right pelvic and para-aortic lymphadenectomy, and omental biopsy. Pathology showed a carcinoid tumor arising in a mature cystic teratoma, confined to the right ovary (FIGO stage IA). Immunohistochemistry confirmed neuroendocrine differentiation (synaptophysin, chromogranin positive). Postoperatively, chromogranin A normalized, serotonin decreased, and proBNP improved. Three months later, she underwent successful bioprosthetic tricuspid valve replacement with ongoing Cardiology follow up.

**Discussion:**

This case illustrates that ovarian carcinoid tumors may present with minimal symptoms despite significant cardiac involvement. Early cardiac evaluation is warranted in suspected carcinoid tumors to identify carcinoid heart syndrome. Overall survival is generally favorable for early stage ovarian carcinoid tumors. Long-term cardiac prognosis is determined by right ventricular function and presence of residual tumor after resection. Management requires individualized and multidisciplinary surgical planning, balancing tumor resection with timely cardiac intervention.

## Highlights

• In ovarian carcinoid tumors, significant cardiac disease may occur with minimal symptoms.
• Survival is excellent for early stage disease, and cardiac prognosis is determined by RV function and residual tumor.
• Careful timing of oncologic and cardiac surgery must balance tumor resection and cardiac optimization.
• Valve replacement is the cornerstone of cardiac intervention, and the role of somatostatin analog therapy is less clear.

## 1 Introduction

Primary ovarian carcinoid tumors are rare, indolent, neuroendocrine tumors representing less than 0.1% of ovarian neoplasms and 1% of all carcinoid tumors (Ahmad et al., 2024). They can arise as a component of mature cystic teratomas or de novo. Approximately 30% of patients with ovarian carcinoid tumors can present with carcinoid syndrome due to secretion of vasoactive substances, like serotonin, histamine, and tachykinins, with predominant symptoms of cutaneous flushing, secretory diarrhea, and bronchospasm (Kulke & Mayer, 1999). Given direct ovarian venous drainage into the systemic circulation, secreted substances bypass hepatic metabolism and can produce carcinoid syndrome in the absence of metastatic liver disease (Fox & Khattar, 2004)..

Of patients with carcinoid syndrome, up to a quarter may have cardiac involvement (Preda et al., 2018). Carcinoid heart syndrome is characterized by fibrotic, mostly right sided, valvular lesions that are thought to arise secondary to serotonin’s stimulatory effect on endocardial collagen (Grozinsky-Glasberg et al., 2022). Cardiac lesions indicate poor prognosis for patients with carcinoid syndrome, with associated valvular dysfunction and heart failure significantly impacting overall morbidity and mortality (Steeds et al., 2019).

We present the case of a patient who was diagnosed with a large adnexal mass in the setting of new onset hypertension and severe tricuspid insufficiency, with elevated serotonin and chromogranin A levels, concerning for ovarian carcinoid tumor causing carcinoid heart syndrome.

## 2 Case presentation

### 2.1 Presentation

**Fig. 1:**
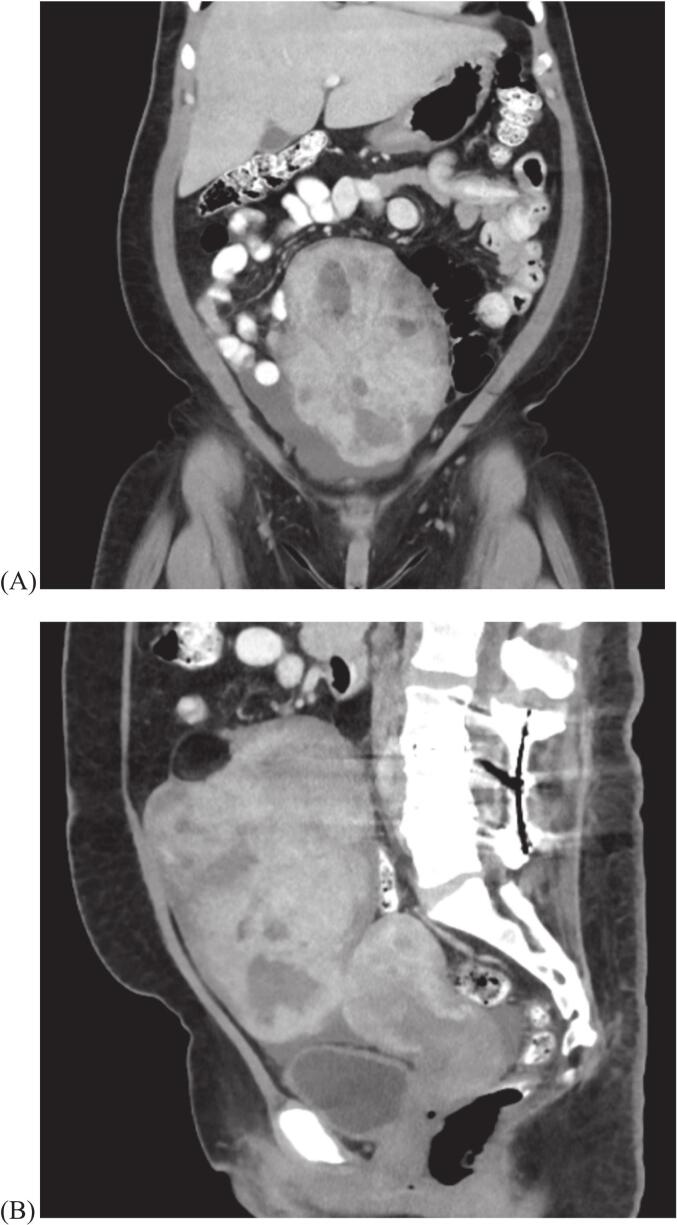
(A) Coronal Computed Tomography scan showing a heterogeneous, predominantly solid, right adnexal mass. (B) Sagittal Computed Tomography scan showing a heterogeneous, predominantly solid, right adnexal mass with fatty subcapsular component.

A 46-year-old G0 female presented to her annual primary care visit with abdominal discomfort and lower extremity swelling. A large, non-tender abdominal mass was palpated on exam. Computed Tomography revealed a heterogeneously enhancing, predominantly solid mass in her right adnexa, measuring 16.8 x 11.9 x 11.5 cm, with fatty and fluid components, suspicious for neoplasm​ (Fig. 1)​. Imaging also demonstrated heterogenous enhancement of her uterus with a 3 cm partially calcified mass in the posterior fundus, a small amount of pelvic ascites, and nonspecific mild enlargement and heterogeneity of the liver without enhancing or suspicious lesions.

In terms of tumor markers, her CA125 level was mildly elevated (92 units/mL), whereas inhibin B (5 pg/mL), CEA (3.3 ng/mL), CA 19–9 (14 units/mL), AFP (1.9 ng/mL), and hCG (<0.6 mIU/mL) levels were within normal limits. Of note, she had a recent colonoscopy with findings of hyperplastic rectal polyps and no other pathologic abnormalities.

**Fig. 2:**
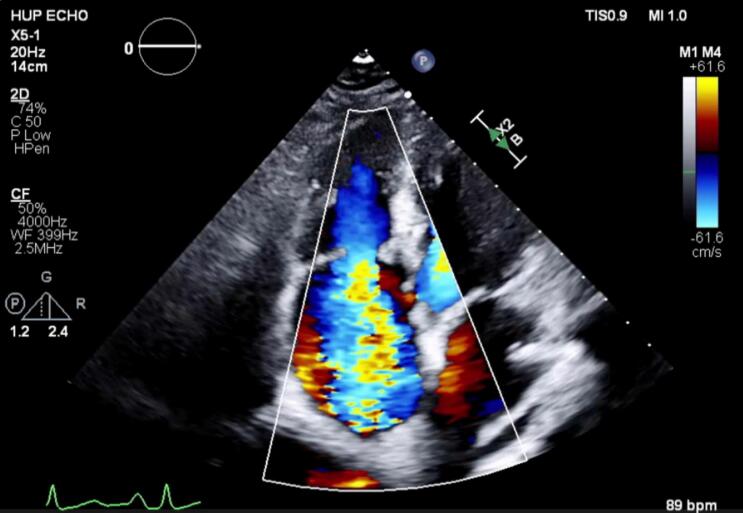
Transthoracic echocardiogram 4-chamber apical view with color Doppler flow demonstrating torrential tricuspid regurgitation.

In the weeks leading up to diagnosis of her adnexal mass, the patient was also diagnosed with new onset hypertension and started on two anti-hypertensive agents with improved blood pressure control. As part of her cardiac workup, she underwent a transthoracic echocardiogram which demonstrated wide open, torrential tricuspid regurgitation in the setting of thickened tricuspid valves with shortened, immobile tricuspid leaflets with no coaptation. Her echocardiogram also noted a left ventricular ejection fraction of 55–60 %, grade 1 diastolic dysfunction, normal right ventricle size and function, and trace to mild pulmonic regurgitation (Fig. 2).

She was evaluated by Cardio-Oncology due to her cardiac findings concerning for carcinoid heart syndrome. The patient disclosed a remote history of flushing with alcohol use but no recent symptoms of flushing, diarrhea, or bronchospasm. She further denied dyspnea, chest pain, subjective dysrhythmia, or other cardiovascular functional limitations. On cardiac exam, she had an elevated JVP at 10–12 cm H_2_O with prominent “v” or venous filling wave as well as a 2/6 systolic murmur, all consistent with tricuspid regurgitation. Her lower extremity edema had resolved by the time of her Cardio-Oncology evaluation. Additional bloodwork established elevated baseline levels of serum serotonin (1646 ng/mL), chromogranin A (281 ng/mL), and N-terminal pro-B-type natriuretic peptide or (NT proBNP, 130 pg/mL). Other differential diagnoses considered for her valvular disease included congenital anomalies, pulmonary hypertension, or blunt trauma; however, in the absence of supporting historical factors and given the constellation of biomarker, cardiac, and abdominopelvic findings, carcinoid heart disease in the setting of a primary ovarian carcinoid tumor was deemed most likely.

The patient’s preoperative cardiovascular risk was acceptable for surgical management of her adnexal mass with planned valvular intervention following surgical recovery.

### 2.2 Surgical management

The patient underwent an exploratory laparotomy, total abdominal hysterectomy, bilateral salpingo-oophorectomy, right pelvic and *para*-aortic lymph node dissection, and omental biopsy. Intraoperatively, yellow, murky ascitic fluid and a large pelvic mass arising from her right adnexa were encountered. Her omentum, bowel, and peritoneal and diaphragmatic surfaces appeared smooth. Her uterus, left ovary, and bilateral fallopian tubes appeared normal, and no enlarged lymph nodes were palpated. Frozen section diagnosis of high grade neoplasm, favoring carcinoma, influenced the intraoperative decision to perform additional staging procedures including contralateral salpingo-oophorectomy, ipsilateral lymph node dissection, and omental sampling.

From an anesthesia standpoint, she maintained hemodynamic and respiratory stability throughout the procedure. Somatostatin analogs were not administered perioperatively. Estimated blood loss was 350 mL. The patient had a routine postoperative course and was discharged from the hospital on postoperative day 4 after meeting all postsurgical milestones.

### 2.3 Pathology

Cytology from pelvic washings did not show evidence of malignancy. Surgical pathology revealed a carcinoid tumor arising in a background of mature cystic teratoma, confined to the right ovary. Her uterus, cervix, bilateral fallopian tubes, left ovary, and omentum revealed benign tissue with no specific pathologic changes. There was no evidence of tumor in resected right pelvic and *para*-aortic lymph nodes.

**Fig. 3:**
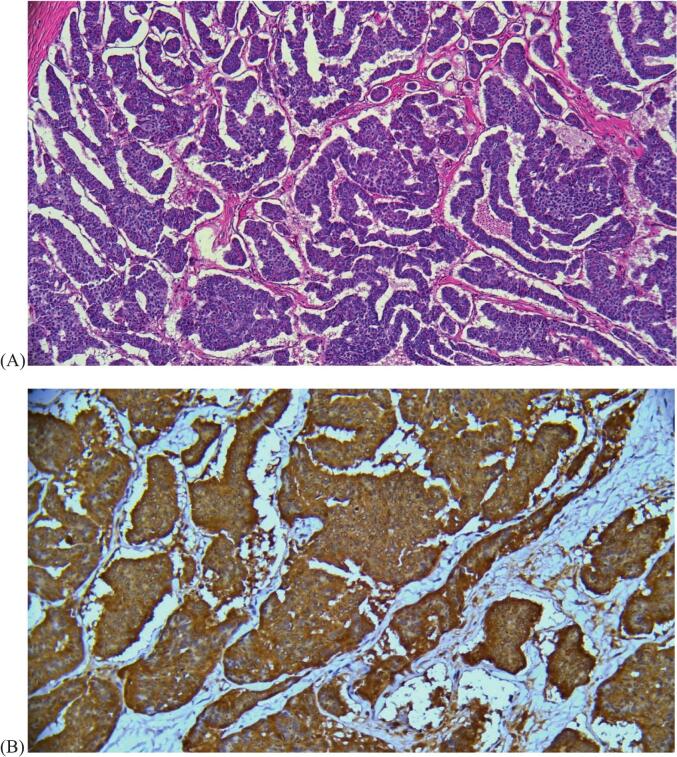

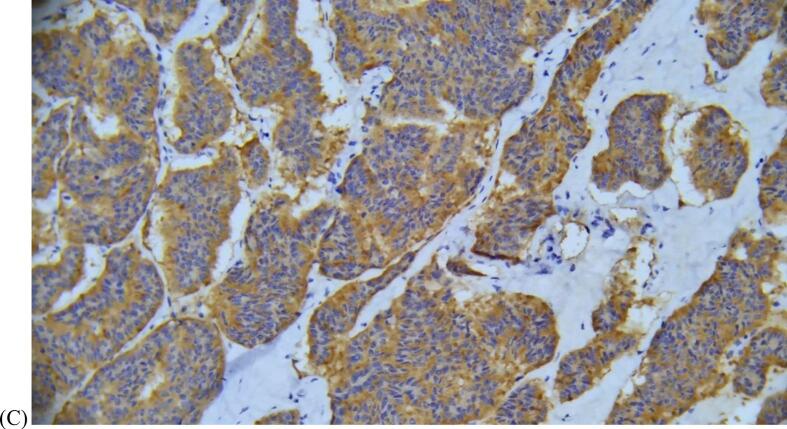
(A) Representative H&E image of the right ovary at 10x magnification. The lesion shows a predominantly insular growth pattern, composed of nests of cells with eosinophilic cytoplasm and round nuclei. The cells demonstrate the salt and pepper chromatin pattern that is characteristic of neuroendocrine tumors. (B) Immunohistochemical stain for chromogranin, a neuroendocrine marker, at 40x magnification showed diffusely positive staining of the right ovary lesion. (C) Immunohistochemical stain for synaptophysin, a neuroendocrine marker, at 20x magnification showed diffusely positive staining of the right ovary lesion.

Immunohistochemistry testing was positive for synaptophysin and chromogranin as well as weakly patchy positive for INSM1 (insulinoma-associated protein 1), consistent with neuroendocrine features (Fig. 3). Stains were negative for TTF1 (thyroid transcription factor) and thyroglobulin, ruling out lung or thyroid tumor origin. The Ki-67 proliferation index was low at <1%.

### 2.4 Follow up

Based on multidisciplinary tumor board discussion, surveillance was recommended for the patient’s International Federation of Gynecology and Obstetrics (FIGO) stage IA ovarian carcinoid tumor. Postoperatively, her serotonin levels demonstrated variable trend: 1646 (preop) to 1756 (POD0) to 1262 (POD3) to 320 (4 weeks postop). Her chromogranin A levels decreased from 281 (preop) to 23 (4 weeks postop). Her proBNP also normalized from 130 (preop) to 65 (4 weeks postop).

### 2.5 Cardiac intervention

Approximately three months after her primary tumor resection, the patient underwent cardiac surgery. Preoperative workup included a repeat transthoracic echocardiogram, re-demonstrating torrential tricuspid regurgitation and normal biventricular function, and Computed Tomography Angiography of her chest, without evidence of metastatic disease or coronary calcifications. The patient’s case was also reviewed by Interventional Cardiology to consider transcatheter valve replacement, however, given the degree of tricuspid valve separation and tethering, she was not a candidate for percutaneous valvular intervention.

She successfully underwent bioprosthetic tricuspid valve replacement and epicardial lead placement, as a prophylactic measure due to high risk of postoperative conduction block, with Cardiac Surgery. Intraoperatively, her pulmonary valve was noted to be anatomically normal without regurgitation or stenosis. She experienced brief episodes of heart block upon weaning from bypass with eventual return of normal sinus rhythm. Her postoperative transesophageal echocardiogram demonstrated normal biventricular function and no leaks in the tricuspid valve replacement gradients. Her immediate postoperative recovery involved diuresis, addition of a beta blocker to her anti-hypertensive regimen without further conduction disturbances, and management of new onset diabetes mellitus. The patient continues to follow closely with Cardiac Surgery and Cardio-Oncology.

## 3 Discussion

We present the case of a patient with ovarian carcinoid tumor complicated by carcinoid heart syndrome who initially came to medical attention with abdominal discomfort and minimal cardiac disease manifestations, namely hypertension and lower extremity edema. Our report is contextualized in existing literature outlined in Table 1, which summarizes 23 cases of primary ovarian carcinoid tumors and carcinoid heart disease between 2020 and 2025. Compared to other reports, the clinical presentation in our case was relatively nonspecific, indicating that patients with ovarian carcinoid tumors may present with complaints outside of classic carcinoid syndrome symptoms (Li et al., 2022). Despite harboring severe tricuspid valvular disease, our patient also exhibited relatively few signs and symptoms of heart failure, which increases risk for delayed recognition until advanced disease progression (Martins Fernandes et al., 2025). Elevated blood pressure may be one of the only early signs of carcinoid disease, as Martins Fernandes et al. similarly described a patient whose initial symptomatic manifestation of ovarian carcinoid tumor was secondary hypertension (Martins Fernandes et al., 2025). These subtleties underscore the importance of maintaining a high index of suspicion for carcinoid tumors and incorporating cardiac evaluation into this workup, even in the absence of overt cardiac symptoms.

**Table 1:** Case reports and series of primary ovarian carcinoid tumors and carcinoid heart disease between 2020 and 2025.

Authors, Year	Age	Symptoms	Exam	Diagnosis	Management	Follow Up
(Franko & Berger, 2000)	72	Dyspnea, peripheral edema, intermittent chest pain, abdominal distention	LE edema, signs of RHF and TR	TTE: severe TR, mildly reduced RV function. Elevated 5-HIAA.	TAH, RSO followed by TVR, CABG.	1 year: asymptomatic
(McMurray, 2000)	57	Diarrhea, orthopnea, LE edema	LE edema, signs of RHF and TR	TTE: TR. Octreoscan: pelvic cul de sac uptake. Elevated 5-HIAA.	TAH, BSO.	Cardiac status stable
(Brunaud et al., 2001)	68	Weight loss	NR	TTE: grade IV tricuspid insufficiency. Elevated serotonin. CT: right ovarian mass.	Bioprosthetic TVR followed by RSO 1 month later.	3 years: no recurrence
(Chaowalit et al., 2004) (case series)	25–73	Symptoms of RHF	NR	TTE: thickened, retracted TV leaflets, dilated right heart chambers. Elevated 5-HIAA levels. Octreoscan: pelvic mass uptake.	All cases: surgical resection of ovarian tumor, right sided valve replacement. Sequence of interventions NR.	12 years longest: no recurrence
(Shapiro & Hanon, 2005)	82	Diarrhea for 1 week, found unresponsive in nursing home	Holosystolic murmur, abdominal mass, pitting edema	CTAP: large pelvic mass. TTE: severe TR. 5-HIAA and chromogranin.	Octreotide for carcinoid crisis. Death from sepsis secondary to pneumonia.	Postmortem diagnosis of ovarian carcinoid tumor, fibrotic TV and PV
(Bonaros et al., 2007)	73	Dyspnea, flushing for 6 months	JVD, lip cyanosis, systolic murmur	TTE: severe TR. Elevated chromogranin A, 5-HIAA. CT: left ovarian mass.	LSO followed by bioprosthetic TVR 10 months later.	NR
(Nyktari et al., 2007)	66	Abdominal fullness, peripheral edema, fatigue for 1 year	NR	TTE: severe TR, PR, mild PS, preserved RV function.	TAH, BSO for ovarian tumors followed by TVR 6 months later.	NR
(Takahashi et al., 2009)	52	Fatigue, dyspnea	Peripheral edema, hepatomegaly	TTE: severe TR, PR. Cardiac cath: elevated right sided pressures. Elevated 5-HIAA. CT: right ovarian tumor.	Bioprosthetic TVR, PVR followed by TAH, BSO 6 months later.	Biomarker resolution
(Aggeli et al., 2010)	60	Fatigue, edema, anorexia for 1 year	HTN, holosystolic murmur, JVD, peripheral edema, hepatomegaly	TTE: severe TR, PR, mildly impaired RV systolic function. Elevated 5-HIAA, proBNP. CT: ovarian mass.	Ovarian tumor resection. TVR, PVR not recommended due to impaired RV function.	NR
(Roberts et al., 2011)	53	DOE, peripheral edema, palpitations (atrial fibrillation)	NR	TTE: thickened MV, AV cusps. Cardiac cath: pulmonary hypertension.	Mechanical AVR, MVR, TV annuloplasty. Re-presented with worsening dyspnea, TTE: dysfunctional RV, effusions. Cardiac arrest and death.	Postmortem diagnosis of carcinoid tumors in right ovary, liver, bowel serosa, uterus, adrenal glands, pacnreas, heart.
(Shin et al., 2011)	60	LE edema, DOE, fatigue for 3 months	Holosystolic murmur, edema, facial flushing, telangiectatic skin changes in face and legs	TTE: severe TR, moderate PR. Elevated 5-HIAA. CT: right ovarian mass.	Ovarian tumor resection. Adjuvant carboplatin, paclitaxel. Patient deferred cardiac surgery due to improved symptoms with medical therapy.	3 years: clinically stable
(Buda et al., 2012)	78	Dyspnea, peripheral edema for 2 months	Holosystolic murmur	TTE: pericardial effusion, severe TR, mild to moderate AS. CT: pelvic mass, ascites. PET/CT: F-18 FDG pelvic mass uptake.	TAH, BSO (FIGO stage IA) followed by hospitalization 3 weeks later for endocarditis, sepsis, not suitable for valve surgery	Death during hospitalization
(Amano et al., 2013)	67	Abdominal distention, RHF	NR	Initial diagnosis with US: ovarian tumor, elevated 5-HIAA. Recurrence 13 years later with CT: *para*-aortic abdominal mass. TTE: severe TR, mild AR.	Primary treatment: 1 cycle of intraperitoneal chemo, 3 cycles of systemic chemo followed by TAH, BSO. Recurrence: tumor resection − lymph node metastasis. No cardiac surgery.	13 years: recurrence
(Damen, 2014)	69	Peripheral edema, dyspnea, abdominal distention for 6 months	Pelvic mass, peripheral edema, JVD	US, CT: left adnexal mass, ascites. Elevated proBNP, chromogranin A, CA125, normal 5-HIAA. TTE: severe TR, mild AR, MR, moderate PR and worsening RV function. Cardiac MRI: carcinoid cardiomyopathy. Octreoscan: pelvic mass uptake.	Bioprosthetic TVR, PVR followed by BSO, omentectomy, bowel and rectal serosal biopsies where mass was adherent, appendectomy (hx prior TAH) a couple months later.	Symptom resolution; monthly CA125, chromogranin A, neuron-specific enolase
(Agarwal et al., 2015)	75	Diarrhea, anorexia for 5 years, acute DOE	NR	Preop TTE: severe TR, PS, PR. Elevated 5-HIAA, serotonin, chromogranin A, pancreastatin, neuron-specific enolase. Octreoscan: pelvic mass uptake.	TVR, PVR followed by ovarian tumor resection 3 months later.	Continued symptom resolution
(Tsugu et al., 2015)	53	Peripheral edema, fatigue for years	JVD, holosystolic murmur, hepatomegaly, edema	Elevated BNP. TTE: severe TR, PR. CT: ovarian tumor.	Bioprosthetic TVR followed by ovarian tumor resection 2 years later.	1 year: no recurrence
(Kolouch et al., 2016)	77	Weight gain, dizziness, dyspnea, edema, facial flushing, diarrhea for a couple months	Plethoric face, systolic murmur, JVD, hepatomegaly, edema	TTE: severe TR, TS, mild PR, adequate RV function. TEE: PFO. Elevated 5-HIAA, chromogranin A. Tectrotyd scan: left ovary uptake. US, CT: pelvic tumor.	TVR, PFO closure (perioperative somatostatin) followed by radical hysterectomy 2 months later.	3 months: elevated biomarkers, paravertebral recurrence treated with monthly somatostatin. 12 months: NED.
(Saraf et al., 2017)	75	LE edema, diarrhea for 2–3 months, hot flashes, DOE	Adnexal mass	US, CT: pelvic mass. Normal CA125, AFP, elevated 5-HIAA. TTE: torrential TR, reduced RV function. Octreoscan: pelvic mass uptake.	Monthly octreotide. TAH, BSO, omental biopsy (octreotide infusion). No cardiac surgery.	Biomarker resolution, some symptom resolution
(Tadokoro et al., 2017)	73	Diarrhea, leg edema, DOE	JVD, hepatomegaly, ascites	TTE: severe TR, moderate PR, moderate AR, MR, PFO. Elevated 5-HIAA, serotonin. MRI: pelvic mass. Sequential blood sampling: drainage of serotonin from ovarian vein to IVC.	Hx TAH, USO for fibroids − noted ovarian tumor, inoperable due to adhesions. Bioprosthetic TVR, PVR, AVR, PFO closure. Octreotide.	16 months: asymptomatic, continued octreotide
(Mansour et al., 2022)	40	Amenorrhea, hirsutism for 1 year, flushing for 5 years, diarrhea for 6 months, also night sweats, weight loss, DOE	Abdominal mass, systolic murmur, JVD, pedal edema, hirsutism	Elevated testosterone, 5-HIAA. CT: pelvic mass, hepatic congestion. Octreoscan: pelvic mass uptake. TTE: severe TR. Cardiac MRI: severe RA, RV dilation.	TAH, BSO (octreotide for 5 days preop) followed by TVR, PFO closure 4 years later due to progression of cardiac symptoms.	4 years
(Wong et al., 2023)	51	LE edema for 6 months, facial flushing for 2 years, diarrhea for 4 years	Large abdominal mass, peripheral edema	CT: ovarian mass. TTE: severe TR, PR, moderate AR, PFO.	TAH, BSO. Postop PET/CT with metastases, started somatostatin analog. Cardiac surgery deferred given improved TR 15 weeks after tumor resection.	8 months: mild to moderate TR. 2 years: asymptomatic, continued somatostatin.
(Ahmad et al., 2024)	54	Dyspnea, edema for months	JVD, pedal edema, holosystolic murmur	Elevated proBNP, 5-HIAA. TTE: TR, PR. RHC: normal pressures. CT: abdominal tumor. Octreoscan: abdominal mass uptake.	Somatostatin analog. TVR, PVR followed by TAH, BSO (FIGO stage 1A).	Symptom improvement
(Martins Fernandes et al., 2025)	63	Weakness, anorexia, diarrhea for 2 months	De novo hypertension	TTE: severe TR, PV thickening, preserved RV function. Elevated 5-HIAA, chromogranin A, normal CA125. PET/CT: avid pelvic mass. US, MRI: ovarian mass.	Multiple anti-HTN agents. Octreotide monthly preop. TAH, BSO (octreotide perfusion) (FIGO stage 1A) followed by bioprosthetic TVR.	3 years: no recurrence

Abbreviations: 5-HIAA: 5-hydroxyindoleacetic acid. AR: aortic regurgitation. AS: aortic stenosis. AV: aortic valve. AVR: aortic valve replacement. BNP: brain natriuretic peptide. BSO: bilateral salpingo-oophorectomy. CABG: coronary artery bypass graft. CT: computed tomography. DOE: dyspnea on exertion. F-18 FDG: fluorodeoxyglucose. FIGO: International Federation of Gynecology and Obstetrics. HTN: hypertension. IVC: inferior vena cava. JVD: jugular venous distention. LE: lower extremity. LSO: left salpingo-oophorectomy. MR: mitral regurgitation. MV: mitral valve. MVR: mitral valve replacement. NED: no evidence of disease. NR: not reported. PET/CT: positron emission tomography-computed tomography. PFO: patent foramen ovale. PR: pulmonary regurgitation. PS: pulmonary stenosis: PV: pulmonary valve. PVR: pulmonary valve replacement. RA: right atrium. RHC: right heart catheterization. RSO: right salpingo-oophorectomy. RV: right ventricle. TAH: total abdominal hysterectomy. TEE: transesophageal echocardiogram. TTE: transthoracic echocardiogram. TR: tricuspid regurgitation. TS: tricuspid stenosis. TV: tricuspid valve. TVR: tricuspid valve replacement. US: ultrasound. USO: unilateral salpingo-oophorectomy.

With regard to management, surgical resection of the primary carcinoid tumor is often curative for early stage disease. However, the sequence of surgical intervention must be individualized to the patient and severity of cardiac symptoms. In patients with deteriorating right heart function or severe symptoms, cardiac valve surgery may be performed first to optimize functional status before tumor resection (Agarwal et al., 2015, Damen, 2014). In other patients, stabilization of carcinoid syndrome symptoms with debulking of hormone-producing tumor may be necessary to promote hemodynamic stability prior to cardiac intervention (Steeds et al., 2019). Our patient’s excellent functional status and minimal symptom burden despite significant valvular disease supported the decision to proceed with tumor resection first, minimizing delays in oncologic management while monitoring her cardiac status closely.

Fertility preservation was not a consideration in our case, but existing literature supports conservative surgical approaches in appropriately selected patients. A large database study showed that unilateral salpingo-oophorectomy offers equivalent overall survival as more extensive surgery in early stage disease (Nasioudis et al., 2020). Furthermore, lymphadenectomy appears to have limited utility. Multiple retrospective series reported no lymph node involvement in patients undergoing comprehensive staging surgery (Kong et al., 2023, Li et al., 2022). These findings suggest that surgical morbidity can be reduced by avoiding unnecessary lymph node dissection in the absence of suspicious findings.

In our case, intraoperative decision-making to perform additional staging procedures was influenced by frozen section analysis, which favored high grade carcinoma. The diagnostic challenge of ovarian carcinoid tumors on frozen section has been documented, with one recent case series of 15 patients showing a concordance rate of 38 % between frozen section and final pathology (Dai et al., 2025). Clear communication between surgical and pathology teams regarding differential diagnoses and preoperative index of suspicion for carcinoid tumor is essential to help guide careful intraoperative decision-making and reduce surgical morbidity from more extensive procedures.

Intraoperative management must also account for the risk of carcinoid crisis, a potentially life-threatening complication caused by sudden release of vasoactive hormones during surgical manipulation or anesthesia. This can manifest as abrupt hemodynamic instability, bronchospasm, and flushing (Grozinsky-Glasberg et al., 2022). Prophylactic strategies include preoperative and intraoperative administration of octreotide, with adjunctive measures such as intravenous fluids, corticosteroids, and antihistamines (Ahmad et al., 2024, Grozinsky-Glasberg et al., 2022). While our patient maintained hemodynamic and respiratory stability without perioperative somatostatin analog administration, in more symptomatic patients, prophylactic octreotide may be warranted to reduce risk of carcinoid crisis.

While the benefits of somatostatin analogs for carcinoid syndrome symptom relief and crisis prevention are well established, their role in cardiac disease is less clear. Wong et al. presented a rare case of marked improvement in cardiac function after surgical resection of a primary carcinoid tumor and initiation of somatostatin analog therapy, challenging the notion that carcinoid heart syndrome is irreversible (Wong et al., 2023). However, the majority of existing reports have not demonstrated regression of cardiac lesions with tumor resection and medical therapy alone (Steeds et al., 2019). As such, timely valve replacement remains definitive treatment for carcinoid heart syndrome to improve cardiac symptoms and prevent progression of heart failure. Due to the extensive fibrosis and endocardial thickening of the tricuspid valve as a result of carcinoid heart disease, severe distortion and immobility of the leaflets and chordae often preclude percutaneous intervention, in other words, transcatheter valve replacement. Therefore, surgical valve replacement remains the standard of care, also enabling concomitant replacement of diseased pulmonary valves and interatrial shunt defects, which are commonly seen in association with tricuspid regurgitation in carcinoid heart disease, although not in our case (Sawma et al., 2025). Long-term cardiac outcomes are largely dictated by right ventricular function and residual tumor status, as patients with preserved function and absence of residual tumor carry favorable prognosis (Sawma et al., 2025).

Although 5-year overall survival for stage I ovarian carcinoid tumors exceeds 95 %, due to the indolent nature of these tumors, long-term surveillance is necessary as relapses have been reported 8–13 years since initial surgery (Nasioudis et al., 2020). Tumors ≥ 4 cm have been associated with worse overall survival, suggesting that patients with larger tumors, such as ours, may benefit from closer surveillance (Nasioudis et al., 2020). Follow-up strategies should include periodic imaging and biochemical monitoring, with particular attention to early signs of recurrent carcinoid activity or cardiac deterioration.

In summary, ovarian carcinoid tumors can present with subtle or nonspecific symptoms, and significant cardiac involvement may exist even in minimally symptomatic patients. For this reason, early and thorough cardiac evaluation is essential when carcinoid tumor is suspected. Management requires careful sequencing of oncologic and cardiac interventions, balancing the urgency of tumor removal against the need for cardiac optimization. While somatostatin analogs are invaluable in reducing burden of carcinoid syndrome symptoms and preventing carcinoid crisis, their role in reversing carcinoid heart syndrome remains uncertain. Ultimately, survival is excellent for early stage ovarian carcinoid tumors, and timely valve replacement remains the cornerstone of cardiac management with long-term cardiac prognosis determined by right ventricular function and presence of residual tumor. Prompt recognition and multidisciplinary coordination are critical to prevent progression to advanced heart failure and improve outcomes for patients with these rare ovarian tumors and cardiac lesions.

Patient Consent

Written informed consent was obtained from the patient for publication of this case report and accompanying images.

Funding Source

None.

Authorship Contribution

MJ: Writing – original draft, review and editing, conceptualization, and investigation. LMG: Writing – review and editing, conceptualization, supervision. JC: review and editing. NT: Writing – review and editing. JB: Writing – review and editing, conceptualization. LS: Writing – review and editing. NL: Writing – review and editing, conceptualization, supervision.

## CRediT authorship contribution statement

**Minyoung Jang:** Writing – review & editing, Writing – original draft, Investigation, Conceptualization. **Lakeisha Mulugeta-Gordon:** Writing – review & editing, Supervision, Conceptualization. **Joseph Carver:** Writing – review & editing. **Natalie Tupper:** Writing – review & editing. **Julie Barbera:** Writing – review & editing, Conceptualization. **Lauren Schwartz:** Supervision. **Nawar Latif:** Supervision, Conceptualization.

## Declaration of competing interest

The authors declare that they have no known competing financial interests or personal relationships that could have appeared to influence the work reported in this paper.
